# Electroacupuncture Inhibits Visceral Nociception via Somatovisceral Interaction at Subnucleus Reticularis Dorsalis Neurons in the Rat Medulla

**DOI:** 10.3389/fnins.2018.00775

**Published:** 2018-10-30

**Authors:** Lingling Yu, Liang Li, Qingguang Qin, Yutian Yu, Xiang Cui, Peijing Rong, Bing Zhu

**Affiliations:** ^1^Department of Neurology, Tongji Hospital, Tongji Medical College, Huazhong University of Science and Technology, Wuhan, China; ^2^Institute of Acupuncture and Moxibustion, China Academy of Chinese Medical Sciences, Beijing, China

**Keywords:** electroacupuncture, visceral pain, colorectal distension, subnucleus reticularis dorsalis, analgesia

## Abstract

Electroacupuncture (EA) is an efficacious treatment for alleviating visceral pain, but the underlining mechanisms are not fully understood. This study investigated the role of medullary subnucleus reticularis dorsalis (SRD) neurons in the effects of EA on visceral pain. We recorded the discharges of SRD neurons extracellularly by glass micropipettes on anesthetized rats. The responses characteristics of SRD neurons to different intensities of EA (0.5, 1, 2, 4, 6, and 8 mA, 0.5 ms, and 2 Hz) on acupoints “Zusanli” (ST 36) and “Shangjuxu” (ST 37) before and during noxious colorectal distension (CRD) were analyzed. Our results indicated that SRD neurons responded to either a noxious EA stimulation ranging from 2 to 8 mA or to noxious CRD at 30 and 60 mmHg by increasing their discharge frequency at an intensity-dependent manner. However, during the stimulation of both CRD and EA, the increasing discharges of SRD neurons induced by CRD were significantly inhibited by 2–8 mA of EA. Furthermore, SRD neurons can encode the strength of EA, where a positive correlation between current intensity and the magnitude of neuronal responses to EA was observed within 2–6 mA. Yet, the responses of SRD neurons to EA stimulation reached a plateau when EA exceeded 6 mA. In addition, 0.5–1 mA of EA had no effect on CRD-induced nociceptive responses of SRD neurons. In conclusion, EA produced an inhibiting effect on visceral nociception in an intensity-dependent manner, which probably is due to the somatovisceral interaction at SRD neurons.

## Introduction

Visceral pain is one of the most common symptoms in patients with gastrointestinal disorders (Sach et al., 2002). It is usually associated with impaired health-related quality of life and a significant health care burden (Chang, 2004; Spiegel et al., 2004). Electroacupuncture (EA) therapy is an effective analgesic by delivering of electrical current to acupoints via acupuncture needles connected to an electrical stimulator (Zhao, 2008). Many behavioral studies have confirmed that EA stimulation exerts good effects on rats with acute or chronic visceral hyperalgesia (Qi and Li, 2012; Wang et al., 2012). In addition, EA therapy has been proven to be effective in treating visceral pain in long–term follow–up clinical trials (MacPherson et al., 2017). It is generally accepted that EA analgesia is an integrative process of afferent impulses between pain regions and acupoints at convergence neurons and this process involves different levels of central structures, such as spinal dorsal horn (Rong et al., 2005), nucleus tractus solitarius (Liu et al., 2014) and periaqueductal gray (Wang et al., 2014). However, the involvement of other convergence neurons in EA analgesia is still unknown.

Accumulating evidence suggests that subnucleus reticularis dorsalis (SRD), the caudal-most aspect of the medulla, plays an important role in the transmission and modulation of nociceptive information (Villanueva et al., 1996). Neurons within SRD are not only activated exclusively by somatic noxious stimuli (mechanical, thermal, or chemical noxious stimuli) applied to widespread areas of the body (Villanueva et al., 1988, 1990), but also respond to noxious visceral stimuli (Roy et al., 1992). Owing to the widespread nociceptive convergence, SRD neurons might contribute to the processing of visceral nociception. In fact, SRD has been verified as a critical region in the pain-inhibiting effect of diffuse noxious inhibitory controls (DNIC) (Bouhassira et al., 1992; Villanueva and Le Bars, 1995). In order to explore the role of SRD neurons in the effects of EA on visceral nociception, the response characteristics of SRD neurons to different intensities of EA before and during noxious CRD was observed and investigated in the present study.

## Materials and Methods

### Animals Preparations

Thirty eight male Sprague–Dawley rats, weighing 220–280 g, were purchased from the Laboratory Animal Center of China Academy of Military Medical Sciences [License number: SCXK–(Military)–2016–0024]. This study was carried out in accordance with the recommendations of the Guideline on the Humane Care and Use of Laboratory Animals issued by the Ministry of Science and Technology of the People’s Republic of China in 2006. The protocol was approved by the CACMS Animal Ethics Committee (No. 20160218). Rats were housed in standard laboratory conditions under artificial 12 h light/dark cycle and at an ambient temperature of 22 ± 0.5. Food and water were available *ad libitum*. After an overnight fast of 12 h, rats were deeply anesthetized with 10% urethane (1.0–1.2 g/kg) and artificially ventilated through a tracheal cannula. Body temperature was maintained at 37 ± 0.5 by means of a feedback controlled homoeothermic heating blanket system.

### Colorectal Distension

Visceral nociceptive stimulus was generated by noxious CRD. Briefly, A 6 cm balloon was gently inserted into descending colon at 4 cm depth through the anus. During the recording sessions, the balloon in the colon was consecutively inflated with air to produce pressure and the intracolonic pressure was monitored with a pressure transducer. The pressure of CRD stimulation applied to rats was 30 and 60 mmHg. In order to prevent possible sensitization triggered by overstimulation of the colorectum, the interval between two CRD stimulations was at least 10 min.

### Recordings

Rats were mounted in a stereotaxic frame with the head fixed in a ventroflexed position by means of a metallic bar cemented to the skull. The caudal medulla was exposed by removing the overlying musculature, atlantooccipital membrane, and dura mater.

Unitary extracellular recordings were made with glass micropipettes (8–12 MΩ) filled with a mixture of 2% pontamine sky blue dye and 0.1 M of natrium aceticum. Micropipettes were inserted on the right side of the medulla, 1.0–2.0 mm caudal to the obex, and 0.5–l.5 mm lateral to the midline.

Single unit activities were fed into a window discriminator and displayed on an oscilloscope screen. The output of the window discriminator and amplifier were fed into a data acquisition system developed by ADInstrument (Power Lab) through a personal computer and Chart 5.0 was used to compile histograms and waveform files for further analysis.

### Electroacupuncture

The rats were treated with EA via a pair of non-insulated acupuncture needles. The needles were inserted into the skin 3 mm apart at the right side of the acupoints “Zusanli” (ST36) and “Shangjuxu” (ST37), which is located in the ipsilateral side of the inserted location of micropipettes. The needles were then connected to an electrical stimulator (88–102G, Nihon Kohden). During a 30 s EA session, intensities of 0.5, 1, 2, 4, 6, and 8 mA were applied in random order. The duration and frequency of electrical stimulation were set at 0.5 ms and 2 Hz.

### Experimental Procedure

(1) After locating a neuron with stable discharges, noxious (pinch) and innocuous (brush) skin stimuli were used to identify the targeted neurons. Since SRD neurons were excited by noxious stimulation to widespread areas of the body including foot, tail and finger, but did not response to innocuous stimuli, we first observed neuronal response to pinch and brush stimulation to foot, tail and finger to identify SRD neurons. Only the neurons that can be excited by pinch stimulation to widespread areas of the body characterized as SRD neurons and were used for further study.

(2) Second, responses of SRD neurons to different intensities of single EA stimulation were compared. The baseline activity was recorded for 5 s, then followed by 10 s EA stimulation, then another 5 s recovery of neuronal discharge was recorded after EA stimulation had stopped.

(3) Third, responses of SRD neurons to graded intensities of CRD stimulation were observed. The baseline activity was recorded for 10 s, followed by 10 s CRD stimulation, then another 10 s of recovery of neuronal discharge was recorded after CRD had stopped.

(4) Fourth, the responses of SRD neurons to different intensities of EA during CRD were observed. A standard conditioned recording procedure was administered for 60 s. Recording of 5 s baseline activities and 5 s recovery neuronal activities were acquired before and after 50 s CRD recording procedure. During the CRD recording procedure, an initial 10 s response of neuron activities to CRD were recorded, 30 s of EA stimulation was administrated and the response of neurons to both EA and CRD was recorded, followed by recording of 10 s of responses to CRD.

**FIGURE 1:**
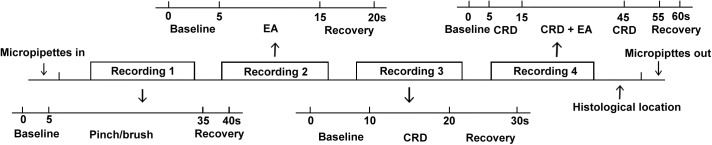
Timeline of experimental prodedure.

The interval between any two stimulations was at least 10 min. The timeline of recording protocol was presented in Figure 1.

### Histological Location

After single unit recordings, the recording sites were marked by electrophoretic deposition of pontamine sky blue and checked by HE coloration. Locations of the recording sites were then determined with reference to the rat brain atlas.

### Data Collection and Statistical Analysis

Neuronal discharges per second (identified as 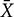 ± SE%) were calculated with PowerLab, Chart 5.0, and SPSS13.0. One way ANOVA and linear regression analyses were used for statistical purposes. *P* < 0.05 was deemed statistically significant.

## Results

### General Characteristics of SRD Neurons on Medulla

**FIGURE 2:**
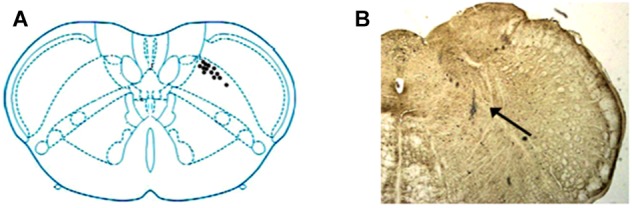
Location of SRD neurons in the medulla. **(A)** Locations of recording sites were marked according to the brain atlas of the rat. **(B)** An individual example showing the location of an SRD neuron marked by pontamine sky blue. Blue dye represents the sites, as indicated by an arrow.

A total of 68 units were recorded within medulla, among which 82.35%(56/68) were characterized as SRD neurons with a “whole body receptive field.” Other neuronal types, such as spinal nucleus of trigeminal, were not considered in this paper. Examination of the rat brain slices verified that the recording sites (*n* = 12) were located in the dorsomedial part of the SRD (Figure 2).

**FIGURE 3:**
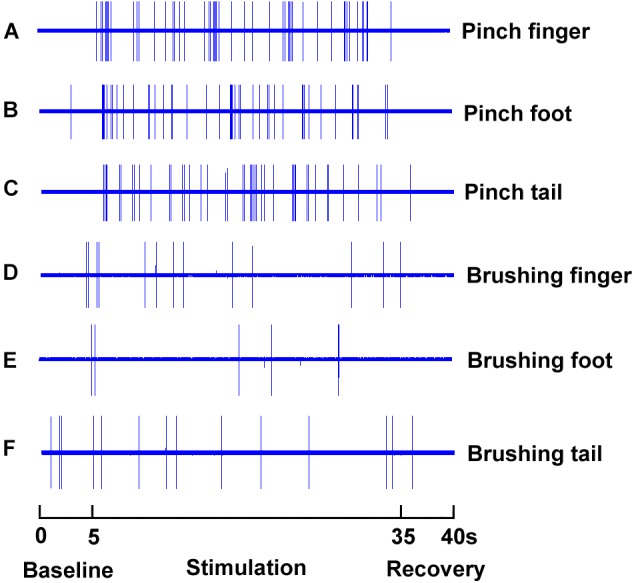
Response characteristics of SRD neurons to innocuous and noxious stimuli. SRD neurons increased neuronal discharges to noxious pinch stimuli of the finger **(A)**, foot **(B)**, and tail **(C)**, but they did not response to innocuous brush stimuli of the finger **(D)**, foot **(E)**, and tail **(F)**.

SRD neurons could be characterized by their response to mechanical and electrical stimuli, our initial approach is to identify these neurons by their responses to pinch and brush stimuli at various part of the rat’s body, which was defined as conditional noxious and innocuous mechanical stimuli. As presented in Figure 3, SRD neurons that were recorded responded to noxious pinch stimulation of the foot, tail and finger with increasing neuronal discharges (Figures 3A–C), but did not respond to innocuous burshing stimulation of the foot, tail and finger (Figures 3D–F).

### Effects of EA With Different Intensities on SRD Neurons Before CRD

**FIGURE 4:**
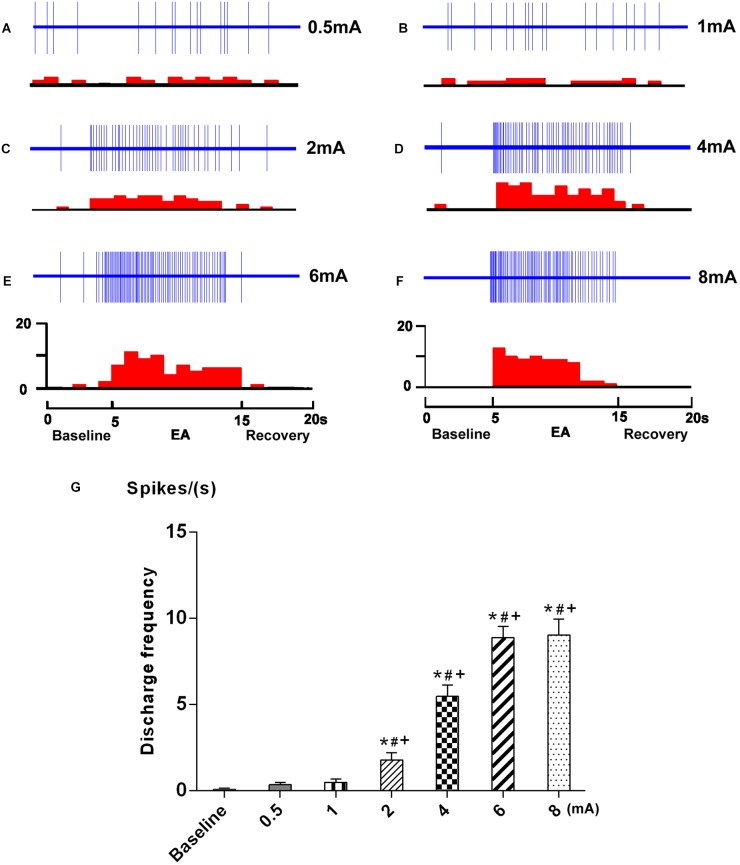
Response characteristics of SRD neurons to graded EA. **(A–F)** Representative examples showing the responses of SRD neuron to graded EA stimulation. The upper rows show the unit discharges at different stimulations and the lower rows show these discharges in histogram. EA (Electroacupuncture) **(G)** A histogram showing the dicharges of SRD neurons (*n* = 7) were significantly activated by EA at 2–8 mA. ^∗^*p* < 0.05 compared with baseline; ^#^*p* < 0.05 compared with 0.5 mA EA; ^+^*p* < 0.05 compared with 1 mA EA.

The neuronal response to graded EA stimulation at acupoints ST 36 and ST 37 was observed on 7 SRD neurons. An individual example of neuronal discharges evoked by graded intensity of EA stimulation is presented in Figure 4. We found that SRD neurons did not respond to low intensity of EA stimulation (0.5–1 mA), but were significantly activated by high intensity of EA stimulation (2–8 mA). After EA stimulation, the average discharge frequency of SRD neurons was significantly increased from 0.08 ± 0.06 spikes/s at baseline to 1.78 ± 0.42 spikes/s (2 mA), 5.47 ± 0.65 spikes/s (4 mA), 8.89 ± 0.65 spikes/s (6 mA) and 9.03 ± 0.92 spikes/s (8 mA) (*P* < 0.05). There is also a significant difference between the effects of high intensity of EA (2–8 mA) and low intensity of EA (0.5–1 mA) (*P* < 0.05).

Furthermore, SRD neurons increased their discharges linearly when the intensity of current was raised from 2 to 6 mA; Further increased the current beyond 6 mA resulted in a plateau effect on neuronal responses. These observations indicated that SRD neurons responsed to noxious EA stimuli, and could encode the intensity of EA stimuli within a specific range.

### Effects of EA With Different Intensities on the Discharges of SRD Neurons During CRD

**FIGURE 5:**
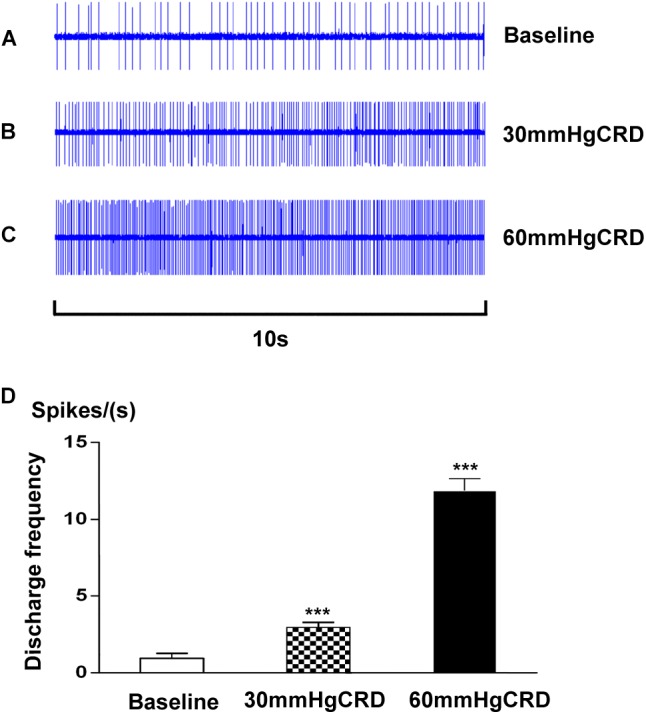
Response characteristics of SRD neurons to graded CRD. **(A–C)** Representative examples showing the responses of SRD neuron to 30–60 mmHg CRD stimulation. **(D)** Cumulative results showing the discharges of SRD neurons (*n* = 7) were significantly activated by 30–60 mmHg CRD stimulation. ^∗∗∗^*p* < 0.001 compared with baseline.

In this experiment, we examined 7 SRD neurons on their reactions to CRD stimulation. As showed in Figure 5, SRD neurons responsed to 30–60 mmHg CRD with increasing discharge rates. After the stimulation of 30 mmHg CRD, the average discharges of SRD neurons were significantly increased from 0.96 ± 0.32 spikes/s at baseline to 2.97 ± 0.32 spikes/s (*P* < 0.001); when CRD was set at 60 mmHg, the average discharges of SRD neurons were increased to 11.87 ± 0.79 spikes/s(*P* < 0.001). This indicates that noxious CRD stimulation could activate the activity of SRD neurons and of significant dose–effect relation.

**FIGURE 6:**
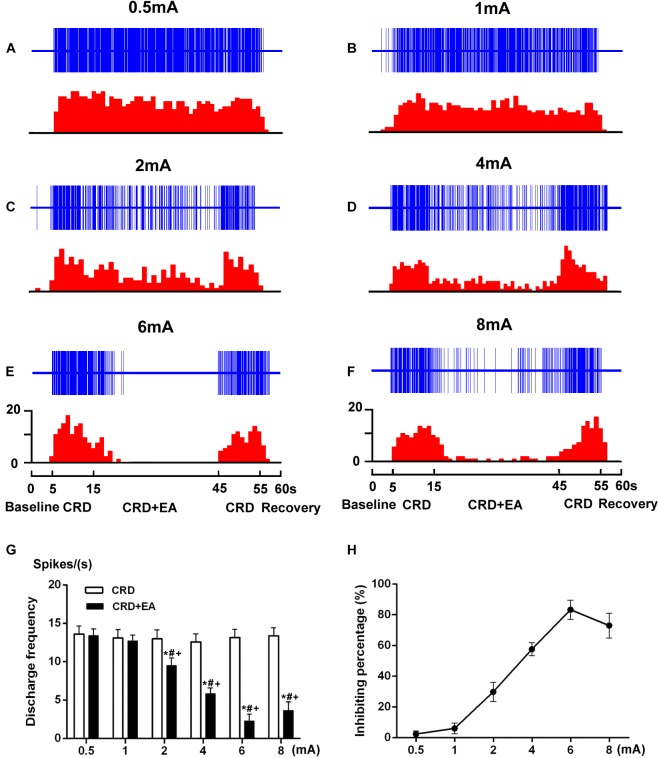
Response characteristics of SRD neurons to different intensities of EA during CRD. **(A–F)** Representative examples showing a decrease in discharges of SRD neurons during the CRD + EA sequence was elicited by EA at 2–8 mA. The upper rows show the unit discharges at different stimulations and the lower rows show these discharges in histogram. Note: CRD (Colorectal distention), CRD + EA (CRD plus EA). **(G)** This histogram shows that CRD induced discharges of SRD neurons were inhibited by EA at 2–8 mA. Data consists of the average spikes per second (mean ± SEM). ^∗^*p* < 0.05 compared with CRD; ^#^*p* < 0.05 compared with 0.5 mA EA; ^+^*p* < 0.05 compared with 1 mA EA. **(H)** This stimulus–response curve shows the percentage of inhibition induced by EA. A positive linear relationship between intensity and inhibition percentage of inhibition was observed within 2–6 mA (*Y* = 10.79 log X + 3.387, *P* < 0.001). Further increased the intensity to 8 mA induced a significant decrease in inhibition percentage.

During the stimulation of 60 mmHg CRD, the response of SRD neurons to different intensities of EA was observed. As illustrated in Figure 6, the increased discharges of SRD neurons induced by CRD could be inhibited by EA at 2–8 mA. However, low intensity of EA (0.5–1 mA) had no significant inhibitory effect on CRD induced noxious discharges of SRD neurons.

When the intensity of the current was set at 2 mA, EA produced a slight, but significant inhibition on the discharges of SRD neurons. The average discharges decreased from 13.02 ± 1.15 spikes/s of CRD to 9.51 ± 0.98 spikes/s, with an inhibiting percentage of 29.91 ± 6.24% (*P* < 0.05, *n* = 7). There is also a significantly difference between the effects of 2 mA EA and 0.5 mA EA (*P* < 0.05), as well as 2 mA EA and 1 mA EA (*P* < 0.05).

When the intensity of the current was set at 4 mA, EA produced a moderate inhibition, the average discharges of SRD neurons decreased from 12.63 ± 1.02 spikes/s of CRD to 5.84 ± 0.74 spikes/s, with an inhibiting percentage of 57.59 ± 4.25 % (*P* < 0.05, *n* = 7). There is also a significantly difference between the effects of 4 mA EA and 0.5 mA EA (*P* < 0.05), as well as 4 mA EA and 1 mA EA (*P* < 0.05).

When the intensity of the current was set at 6 mA, EA produced a stronger inhibition, the average discharges of SRD neurons decreased from 13.15 ± 1.08 spikes/s of CRD to 2.30 ± 0.88 spikes/s, with an inhibiting percentage of 83.36 ± 6.31 % (*P* < 0.05, *n* = 7). There is also a significantly difference between the effects of 6 mA EA and 0.5 mA EA (*P* < 0.05), as well as 6 mA EA and 1 mA EA (*P* < 0.05).

When the intensity of EA was set at 8 mA, EA still produced a strong inhibition, yet this inhibitory effects of EA on the noxious responses of SRD neurons had reached a plateau. After EA, the average discharges of SRD neurons decreased from 13.39 ± 1.02 spikes/s of CRD to 3.65 ± 1.13 spikes/s, with a inhibiting percentage of 73.00 ± 8.14 % (*P* < 0.05, *n* = 7). Although there is also a significantly difference between the effects of 8 mA EA and 0.5 mA EA (*P* < 0.05), as well as 8 mA EA and 1 mA EA (*P* < 0.05). There is no significantly difference between the effects of 8 mA EA and 6 mA EA.

These results indicated that the noxious responses of SRD neurons to CRD could be inhibited by EA in an intensity dependent manner, but such an inhibiting effect of EA reached a plateau when the current exceeded 6 mA.

## Discussion

Although EA has been practiced in China since the early 1950s and is widely used for analgesia in clinic, the mechanisms of EA analgesia on visceral pain are not fully understood. In this study, we explored the role of SRD in EA analgesia on visceral pain. Our results demonstrated that the activities of SRD neurons were activated by either a single noxious CRD or 2–8 mA of EA stimulation by increasing their spontaneous discharges. However, the increased discharges of SRD neurons resulted from the stimulation of noxious CRD could be inhibited by 2–8 mA of EA stimulation. These results suggest that visceral nociception could be inhibited by EA via somatovisceral interaction onto SRD neurons.

The antinociceptive effects of EA on visceral nociception were observed on acupoints ST 36 and ST 37. According to traditional acupuncture theory, the two acupoints are lower confluent acupoint of the meridians of stomach and large intestine, and are used for the treatment of gastrointestinal disease. It has been confirmed by animal experiments that EA and acupuncture stimulation applied at acupoints ST 36 and ST 37 exerts good effects on rats with acute visceral hyperalgesia induced by acetic acid (Qi et al., 2016, 2018) and CRD (Rong et al., 2005). In addition, ST 36 has a specific effect on CRD–induced changes in blood pressure, abnormal electrogastrogram and gastric tension (Chen et al., 2011). In the present study, we observed that noxious EA stimulation of ST 36 and ST 37 is effective in inhibiting visceral nociception at SRD level. Together with previous studies, these findings provide evidence for the efficacy of ST 36 and ST 37 in treating visceral gastrointestinal pain.

SRD could play a specific role in processing nociceptive information. It was a well delimited area within the caudal most aspect of the medulla. Previous electrophysiological studies had clearly demonstrated the response properties of neurons within SRD to somatic and visceral inputs. The great majority of neurons within SRD did not exhibit spontaneous activity, but these neurons were activated exclusively by thermal, mechanical, and electrical noxious stimulation on any part of the body surface, thus exhibiting a “whole body” receptive field (Villanueva et al., 1988, 1990). In addition, SRD neurons also responded to noxious visceral stimulation (Li et al., 2013; Yu et al., 2014). Similarly, the present study observed the activation responses of SRD neurons evoked by noxious pinch stimulation applied to the foot, tail and finger, and also by noxious CRD stimulation and high intensity of EA stimulation within 2–8 mA. However, SRD neurons did not response to innocuous brush stimulation nor to low intensity of EA stimuli within 0.5–1 mA. Interestingly, the threshold of the intensity for Aδ–and C–fiber activation evoked by EA stimulation was approximately 1.68 ± 0.53 mA and 4.78 ± 0.45 mA, respectively (Zhu et al., 2004). Therefore, EA with the intensity of 2–8 mA could be identified as noxious stimulation. This indicated that SRD neurons have similar characteristics of responses to EA stimulation as to other somatic stimulation, receive solely noxious information.

EA achieves analgesic effects on visceral pain via somatovisceral interactions between pain regions and acupoints at different level of central nervous system. The inhibition of visceral nociception induced by acupuncture has been observed at spinal wide dynamic range neurons in our previous study (Rong et al., 2005). However, the inhibition was abolished by blockade of the central descending pathway, indicating that the effects of EA on visceral nociception may not only modulate by spinal level, but many others in supraspinal center. Accumulating evidences showed that the occurrence of a reciprocal connection between dorsal reticular structure and spinal neurons (Villanueva et al., 1991; Almeida et al., 1993). SRD is an important structure in spino-reticulo-spinal loop, which implicated in the modulating of ascending noxious information (Almeida et al., 1993). In this study, we clearly demonstrated that the nociceptive response of SRD neurons induced by noxious CRD stimulation could be inhibited by EA stimulation within the intensity of 2–8 mA. Because SRD neurons receive only noxious inputs, the nociceptive responses of SRD neurons were not affected by EA stimulation within the intensity of 0.5–1 mA. Our findings provide evidence for the interaction of visceral and EA inputs onto SRD neurons. Together with previous electrophysiological studies (Bing et al., 1991), we reasoned that SRD may be involved in EA analgesia on visceral pain by means of spino-reticulo-spinal feed-back mechanism.

The mechanisms underlying EA induced segmental and extrasegmental analgesia are differ. Electrophysiological studies on somatic pain have shown that segmental analgesia of homotopic EA stimulation can be elicited by the activation of Aβ- and part of Aδ – fibers, whereas extrasegmental analgesia of heterotopic EA stimulation is only effective with intensities strong enough to excite Aδ–or C–fibers (Xu et al., 2003; Zhu et al., 2004; Xin et al., 2016). It is very likely that SRD neurons are involved in the mechanism of the widespread extrasegmental antinociceptive effects of EA.

When the intensity of EA increased to noxious range, EA stimulation produced apparent inhibition of the nociceptive responses in the SRD neurons. The widespread extrasegmental analgesia induced by noxious EA stimuli can also be illustrated by DNIC that was proposed by Le Bars et al. (1979a). DNIC refer to the phenomena that noxious response of convergent neurons of the dorsal horn and/or medullary dorsal horn was inhibited by heterotopic noxious stimuli (Cadden et al., 1983; Cadden and Morrison, 1991). SRD has been verified as an important supraspinal relay in DNIC. Similar to the response properties of SRD neurons, DNIC is triggered only by A δ–and C–fibers (Le Bars et al., 1979b). The arrival of nociceptive inputs to medulla can activate SRD neurons and trigger DNIC function, and then trigger a negative feedback to nociceptive signals (Le Bars, 2002). Actually, 2 Hz EA exert analgesic effect on chronic pain by improve DNIC function (Yuan et al., 2018).

We also found that there is a positive relationship between current intensity and the magnitude of neuronal responses to EA within 2–6 mA, while the responses of SRD neurons to EA reached a plateau beyond 6 mA. The encoding property of SRD neurons to EA closely resembles the encoding property of SRD neurons to electrical stimuli (Villanueva et al., 1989). SRD neurons can encode the intensity of electrical stimuli, especially within the noxious range (Villanueva et al., 1989, Gall et al., 2000), For instance, they responded to the graded intensity of electrical stimuli in the range of 1.5–6.0 mA (Villanueva et al., 1989). However, we must point out that if the electrical current is strong enough to excite C–fibers, EA treatment will inevitably cause unbearable pain in clinical practice. As such, if the intensity of EA exceeds a certain range, this treatment is not suitable for analgesia in patients. The present results show that 6 mA may be sufficient to elicit the optimal analgesic effects on visceral pain rats.

## Conclusion

The present study emphasizes the important role of SRD neurons in EA analgesia on visceral pain. In normal state, the spontaneous activity of SRD neurons could be activated by EA within 2–8 mA. However, during CRD, the nociceptive responses of SRD neurons could be inhibited by EA within 2–8 mA. In summary, we speculated that the transmission of visceral nociceptive could be inhibited by EA via somatovisceral interaction at SRD neurons.

## Author Contributions

LY, LL, and QQ performed the experiments. BZ designed the experiments. LY and YY analyzed the data and drafted the manuscript. All the authors discussed the results, reviewed the final manuscript, and approved it for the publication.

## Conflict of Interest Statement

The authors declare that the research was conducted in the absence of any commercial or financial relationships that could be construed as a potential conflict of interest.

The reviewer ML declared a shared affiliation, with no collaboration, with one of the authors, LY, to the handling editor at time of review.
